# Impaired plasticity of cortical dendritic spines in P301S tau transgenic mice

**DOI:** 10.1186/2051-5960-1-82

**Published:** 2013-12-17

**Authors:** Nadine A Hoffmann, Mario M Dorostkar, Sonja Blumenstock, Michel Goedert, Jochen Herms

**Affiliations:** 1 Center for Neuropathology and Prion Research, Ludwig-Maximilians University, Munich, Germany; 2 Department of Translational Brain Research, German Center for Neurodegenerative Diseases (DZNE), Munich, Germany; 3 Medical Research Council Laboratory of Molecular Biology, Cambridge, UK; 4 Munich Cluster of Systems Neurology (SyNergy), Munich, Germany

**Keywords:** Tau, P301S mutation, Dendritic spines, Synaptic plasticity, Two photon in vivo imaging

## Abstract

**Background:**

Illuminating the role of the microtubule-associated protein tau in neurodegenerative diseases is of increasing importance, supported by recent studies establishing novel functions of tau in synaptic signalling and cytoskeletal organization. In severe dementias like Alzheimer’s disease (AD), synaptic failure and cognitive decline correlate best with the grade of tau-pathology. To address synaptic alterations in tauopathies, we analyzed the effects of mutant tau expression on excitatory postsynapses *in vivo*.

**Results:**

Here we followed the fate of single dendritic spines in the neocortex of a tauopathy mouse model, expressing human P301S mutated tau, for a period of two weeks. We observed a continuous decrease in spine density during disease progression, which we could ascribe to a diminished fraction of gained spines. Remaining spines were enlarged and elongated, thus providing evidence for morphological reorganization in compensation for synaptic dysfunction. Remarkably, loss of dendritic spines in cortical pyramidal neurons occurred in the absence of neurofibrillary tangles (NFTs). Therefore, we consider prefibrillar tau species as causative for the observed impairment in spine plasticity.

**Conclusions:**

Dendritic spine plasticity and morphology are altered in layer V cortical neurons of P301S tau transgenic mice *in vivo*. This does not coincide with the detection of hyperphosphorylated tau in dendritic spines.

## Background

Intracellular aggregates of the microtubule-associated protein tau are found in a large number of neurodegenerative diseases, including AD and frontotemporal dementia and parkinsonism linked to chromosome 17 (FTDP-17) [1,2]. In these so-called tauopathies, hyperphosphorylation of tau promotes its detachment from microtubules, resulting in tau mislocalization to the somatodendritic compartment, where it forms oligomers, neuropil threads and NFTs. The pathological mechanisms triggered by abnormal tau remain largely unknown, especially with regard to synaptic failure. In sporadic AD, tau-pathology precedes deposition of extracellular amyloid-β and correlates best with the grade of dementia [3]. Moreover, synaptic dysfunction is believed to be the primary cause of cognitive decline in AD and other dementias [4]. Thus, for understanding the pathological mechanisms causing tauopathies, as well as for the development of therapies, illuminating the effects of tau on synapses is indispensable.

Recently, Hoover et al. showed that dendritic spines are the locus of early synaptic malfunction caused by tau. Thereby, mistargeting of hyperphosphorylated tau to intact spines mediates synaptic dysfunction independently of neurodegeneration [5]. This detrimental role of hyperphosphorylated and misdistributed tau is further supported by the finding that dendritic tau mediates amyloid-β toxicity [6] and tau knockout prevents early lethality and behavioural deficits in an AD mouse model [7]. Dendritic spines and synaptic pathology in tauopathy mouse models have also been analyzed by other groups, obtaining divergent results: rTg4510 mice, transgenic for human tau with the FTDP-17 mutation P301L, show reduced spine density and impaired dendritic complexity of pyramidal neurons in the cortex, both in the absence and presence of NFTs [8,9]. Synapse loss in the hippocampus before the emergence of fibrillary tangles was described for a mouse line expressing P301S mutated human tau under the murine prion promoter [10]. A reduced hippocampal spine density was also found in Tau_RD_/ΔK280 mice, expressing proaggregation mutant tau [11]. Conversely, an age-dependent increased spine density in cortical layer III, but no alterations in the hippocampus were reported for transgenic mice expressing human P301L or wildtype tau [12]. In cortical layer III pyramidal neurons of mice expressing all six human tau isoforms instead of murine tau, the spine volume decreases with advancing age, while spine density stays unaffected [13]. Since these studies were based on fixed brain tissue, so far, dendritic spine plasticity has not been analyzed in a tauopathy mouse model *in vivo*. Therefore, alterations in the total number of spines could not be attributed to changes in spine kinetics.

In this study, we investigated the effects of mutant tau expression on the structural plasticity of dendritic spines in P301S Tau mice [14]. They express human tau protein bearing an FTDP-17 mutation [15] under the control of the murine *thy1*-promoter. FTDP-17 patients carrying this mutation suffer from an early onset, rapidly progressive frontotemporal dementia and parkinsonism in combination with epileptic seizures [16]. Homozygous P301S Tau mice similarly exhibit severe tau-pathology already at 5–6 months of age: Abundant intracellular filaments composed of hyperphosphorylated tau deposit in several regions of the central nervous system (CNS), especially the brain stem and spinal cord [14,17-20]. Progressive neuron loss, accompanied by neuroinflammation, can be found in cortical layers I/II [21], but early behavioral abnormalities have been reported well before the onset of neurodegeneration [22].

By means of long-term two-photon *in vivo* imaging, we found a progressive spine loss on apical tuft dendrites of cortical layer V neurons in 4-month-old P301S Tau mice. The remaining spines were enlarged in head volume and increased in length. Immunohistochemical characterization of the analyzed pyramidal neurons revealed them to be bare of NFTs or hyperphosphorylated tau. In contrast to other brain regions like hippocampal CA3 pyramidal neurons, where abundant deposits of hyperphosphorylated tau can be found in dendritic spines, these were absent in the cerebral cortex. This observation is in line with what is seen in the brain tissue of AD patients [23,24].

## Methods

### Transgenic mice

For two-photon *in vivo* imaging, homozygous mice transgenic for the 383 amino acid isoform of human tau protein with the familial FTDP-17 mutation P301S [14] were crossbred with mice of the YFP-H line [25] (obtained from The Jackson Laboratory, Bar Harbor, ME, USA). Mice homozygous for tau (P301S Tau x YFP-H mice) were used in experiments, while mice lacking mutant tau served as wildtype controls. The mice were exclusively male and the offspring of the same founder animals. Tau homozygosity was determined by real time PCR. Mice were group housed under pathogen-free conditions until surgery from which on they were singly housed. All procedures were performed in accordance with an animal protocol approved by the Ludwig Maximilian University of Munich and the Government of Upper Bavaria (ref. num. 55.2-1.54-2531-110-06).

### Cranial window surgery

**Figure 1:**
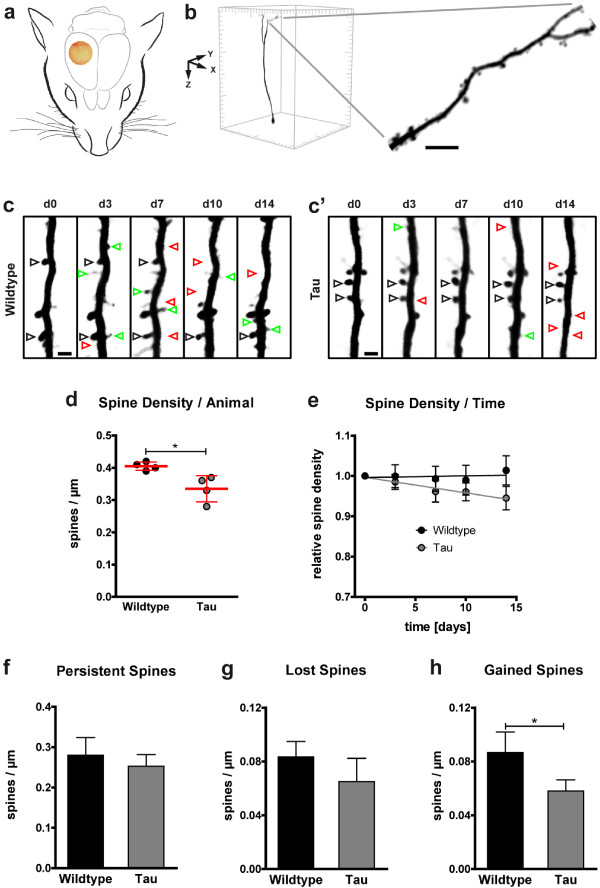
**Decreased dendritic spine density and impaired spine kinetics in P301S Tau mice. a** Location of the chronic cranial glass window, as implanted for long-term *in vivo* imaging of YFP-expressing neurons. **b** Example of an isolated, volume rendered layer V neuron in a 425 × 425 × 550 μm^3^ xyz-stack of the somatosensory cortex. A section of an apical tufted dendrite, stretching in parallel to the brain surface, is shown in higher magnification (maximum intensity projection; scale bar: 5 μm). **c**-**c’** Dendritic elements in 4-month-old wildtype **(c)** and P301S Tau mice **(c’)** were analyzed by high-resolution two-photon *in vivo* imaging over a time period of two weeks (d: day). Repetitive imaging allows discrimination of stable, gained, and lost spines (black, green, and red arrowheads respectively; maximum intensity projections; scale bars: 2 μm). **d** Spine density was reduced in P301S Tau mice at the first *in vivo* imaging session. Presented are means per animal of 8 mice; 61–82 dendrites per group; 14–22 dendrites per mouse; means ± SD per group in red. **e** During the following 14 days, the spine density stayed stable in wildtype mice but further declined in P301S Tau mice. Presented are means ± SEM of 28–41 dendrites in 3–4 mice per group, 8–12 dendrites per mouse; normalized to the first imaging day. Line, linear fit. p <0.01 (F test of means). **f**-**h** In P301S Tau mice, the densities of persistent **(f)** and lost spines **(g)** was not significantly impaired, while the density of gained spines was decreased **(h)**. Presented are means ± SD of 3–4 mice per group; 28–41 dendrites; 8–12 dendrites per mouse. **b**, **c**, **e**, **f** &**g**: Unpaired *t* test; * p < 0.05.

A chronic cranial glass window was implanted over the right hemisphere of the cerebral cortex (Figure 1a), applying the open skull preparation as previously described [26,27]: The mice were anesthetized by an intraperitoneal injection of ketamine/xylazine (130 and 10 μg/g body weight, respectively). Additionally, anti-inflammatory dexamethasone (6 μg/g body weight) was administered intraperitoneally and body temperature was maintained by a heating pad. A circular piece of the skull, 4 mm in diameter, was removed above the somatosensory cortex (centered at the right parietal bone, approximately 2 mm caudal of the bregma and 2.5 mm lateral of the midline), using a dental drill (Schick-Technikmaster C1; Pluradent; Offenbach, Germany). To close the craniotomy, a round coverslip (5 mm in diameter) was glued to the skull using dental acrylic (Cyano-Veneer fast; Heinrich Schein Dental Depot, Munich, Germany). A small metal bar, containing a screw thread, was mounted next to the coverslip to allow fixation and precise repositioning of the mouse head during subsequent imaging sessions. After surgery, mice received a subcutaneous analgesic dose of carprophen (7.5 μg/g body weight Rimadyl; Pfizer, New York, USA).

### Long-term two-photon *in vivo* imaging

To avoid neuroinflammatory responses, *in vivo* imaging was performed after a 3 week recovery period. Each imaging session lasted for no longer than 60 min during which mice were anesthetized by an intraperitoneal injection of ketamine/xylazine (130/10 μg/g body weight) and body temperature was maintained by a heating pad. YFP was excited by a titanium-sapphire laser (MaiTai, Spectra-Physics, Darmstadt, Germany) at 880 nm and the emission was collected using a 500–550 nm bandpass filter (LSM 5MP, Carl Zeiss MicroImaging GmbH, Jena, Germany). Less than 50 mW laser power was delivered to the tissue to prevent laser-induced phototoxicity. A 20x water-immersion objective (1.0 NA; Carl Zeiss MicroImaging GmbH) was used to acquire overview stacks of 425 × 425 × 350 μm^3^, starting at the brain surface, with 0.41 × 0.41 × 3.00 μm^3^ xyz-scaling. Subsequently, single spiny dendritic elements at a depth of 50–120 μm of this volume were imaged at high resolution (0.16 × 0.16 × 1.00 μm^3^ xyz-scaling). To ensure that these apical tuft dendrites arose from layer V neurons, and exclude YFP-expressing layer II/III neurons, only dendrites protruding a depth of 350 μm were chosen (Figure 1b). In subsequent imaging sessions, previously imaged regions were relocalized and precisely aligned based on the unique pattern of blood vessels, neuronal cell bodies, and their processes. Laser intensity was adjusted to keep the emitted YFP fluorescence stable.

### Immunohistochemistry

Brains of 4- and 6-month-old P301S Tau x YFP-H mice of mixed sex were processed for immunohistochemistry. Following transcardial perfusion of the mice with phosphate buffered saline (PBS) and 4% paraformaldehyde (PFA) in PBS at 4°C, brains were removed and fixed in 4% PFA in PBS over night at°C. In an attempt to improve tissue preservation for tau detection in dendritic spines, as described by Kremer et al. (2011), some mice were alternatively perfused with PBS at 4°C, brains were removed and hemispheres were fixed over night at 4°C in either 4% PFA in PBS or in Bouin solution (71.4% saturated picric acid, 23.8% formaldehyde, 4.8% glacial acidic acid). 100 μm free-floating frontal sections of the somatosensory cortex were cut on a vibratome (VT1000S, Leica Microsystems GmbH, Wetzlar, Germany). During all following steps, the sections were kept on a shaker at room temperature. To permeabilize the tissue, the sections were incubated over night in 2% Triton X-100 in PBS.

For AT8-, AT180-, and HT7-stainings, background signal was reduced using Endogenous Biotin-Blocking Kit E-21390 (Molecular Probes, Eugene, OR, USA). Sections were incubated in 10% normal goat serum (NGS), 1% bovine serum albumin (BSA), and 0.1% Triton X-100 in PBS before primary antibody incubation in 5% BSA and 0.1% Triton X-100 in PBS over night. The following monoclonal antibodies, conjugated to biotin, were used in 1:200 dilutions: HT7 recognizing human tau, AT8 recognizing human and murine tau phosphorylated at S202 and T205, and AT180 recognizing human and murine tau phosphorylated at T231 and S235 (all from Thermo Scientific Pierce Protein Research Products, Rockford, IL, USA). Enzyme-mediated antibody-detection was performed using TSA™ kit #26 (Invitrogen, Carlsbad, CA, USA) with HRP-streptavidin (1:200) in 1% BSA in PBS and Alexa Fluor 647 tyramide (1:200), according to the manufacturers’ instructions.

For AT100-stainings, non-specific epitopes were blocked with Casein I-Block (Invitrogen) for 1 hour. AT100 (Thermo Scientific Pierce Protein Research Products), recognizing human and murine tau phosphorylated at Alzheimer-specific epitopes S212 and T214, was applied for 4 hours, diluted 1:200 in PBS. Detection was performed by incubating the sections with secondary anti-mouse antibody conjugated to Alexa Fluor 647 (1:200 in PBS; Invitrogen) for 4 hours.

For all stainings, sections were finally washed 5x10 min with PBS before mounting on glass coverslips using fluorescence mounting medium (Dako, Glostrup, Denmark).

### Confocal microscopy

Fluorescence images were acquired with a confocal laser scanning microscope, mounted on an inverted microscope support (LSM 510 and AxioVert 200, Carl Zeiss MicroImaging GmbH). Alexa Fluor 488 was excited by an argon laser at 488 nm and emission was collected using a 500–550 nm bandpass filter. Alexa Fluor 647 was excited by a helium-neon laser at 633 nm and emission was collected using a 650 nm longpass filter.

For the AT8-YFP-correlation, a 40x oil-immersion objective (Plan-Apochromat, NA 1.3; Carl Zeiss MicroImaging GmbH) was used to obtain 360 × 360 × 50 μm^3^ stacks with 0.35 × 0.35 × 2.00 μm^3^ xyz-scaling. Per animal 10 stacks of layer V neurons were analyzed.

### Image processing and data analysis

Dendritic spine density was determined using ZEN 2011 Light Edition software (version 7.0, Carl Zeiss MicroImaging GmbH). Images were corrected with a gamma of 0.45 and spines were counted manually by scrolling through the z-stacks. In time-series, a dendritic spine was defined as the same if its location did not change within a range of 1 μm along the dendrite. Since z-scaling was limited to 1 μm, only protrusions emanating laterally from the dendritic shaft were analyzed.

For morphological spine analysis, the three-dimensional *in vivo* images were deconvolved (AutoQuant, version X2.0.1, Media Cybernetics, Bethesda, USA) and semi-automatically remodelled (Imaris 6.1.5, Bitplane, Zurich, Switzerland). Spine subtypes were identified using the Imaris XT spine classification module, based on the following hierarchical algorithms (adapted from [28]): mushroom spine: “max_width(head)/min_width(neck) > 1.4 and max_width(head) > 0.4 μm and min_width(neck) > 0 μm”; stubby spine: “length(spine)/mean_width(neck) ≤ 3 or min_width(neck) = 0 μm or > 0.5 μm”; thin spine: length(spine)/mean_width(neck) > 3.

Analysis was performed blinded in respect to mouse genotype.

YFP- and / or AT8-positive layer V pyramidal neurons were counted manually using ZEN 2011 Light Edition software (version 7.0; Carl Zeiss MicroImaging GmbH).

All data are presented as mean ± SD or ± SEM, as stated in the figure legends. Statistical differences between two groups were determined using unpaired *t* test. Since spine head volumes had a log-normal distribution, in this exceptional case the *t* test was done on log-transformed data. All other measurements represent means of means, which according to the central limit theorem approximate a normal distribution. Therefore, we used a parametric test. The slope from a linear regression was tested for statistical difference from zero by *F* test. Statistical analysis and graphs were done using Prism software (version 5.04, GraphPad Software Inc., La Jolla, CA, USA). Figures were arranged using Adobe Illustrator CS4 Extended software (version 11.0.2, Adobe Systems, San Jose, CA, USA).

## Results

In order to analyze the effects of cortical tau-pathology on dendritic spines, we performed long-term two-photon *in vivo* imaging of P301S Tau mice (Figure 1a). The line was crossbred with mice of the YFP-H line, expressing yellow fluorescent protein in a subset of cortical neurons, i.e. layer II/III and V pyramidal neurons (Figure 1b). Thus, we were able to follow the fate of individual dendritic spines in 4-month-old homozygous P301S Tau mice and age-matched wildtype mice through a chronic cranial window (Figure 1c-c’).

### Decreased cortical spine density in P301S tau mice

The spine densities of apical tuft dendrites of layer V neurons showed high variance, ranging from 0.24 to 0.60 spines/μm in wildtype mice and 0.20 to 0.60 spines/μm in P301S Tau mice. Overall, we found a reduction in the mean spine density of P301S Tau mice by 17% (0.34 ± 0.04 compared to 0.41 ± 0.01 spines/μm in wildtype mice, Figure 1d). By relocalizing and repeatedly imaging the same dendritic elements every 3–4 days for a period of two weeks (Figure 1c-c’), we observed further aggravation of total spine loss in P301S Tau mice during disease progression: The relative spine density declined to 0.94 ± 0.15 of the initial value, while remaining unchanged in wildtype mice (1.01 ± 0.13; Figure 1e).

### Tau transgene expression affects spine kinetics

High-resolution, long-term *in vivo* imaging enables very detailed analysis of spine kinetics (Figure 1c-c’). By carefully following the fate of single spines, the densities of stable, gained, and lost spines can be determined for each consecutive imaging session, providing a measurement of synaptic plasticity. We detected no significant change in the densities of persistent spines (i. e. stable for at least one week; Figure 1f) and lost spines (i. e. disappearing from one to the consecutive imaging session; Figure 1g) in P301S Tau mice. However, while in wildtype mice the permanent loss and gain of spines was well-balanced (0.083 ± 0.012 vs. 0.087 ± 0.015 spines/μm, respectively; Figure 1g-h), the spine turnover in P301S Tau mice was shifted: The density of gained spines (i. e. newly emerging from one to the consecutive imaging session) was reduced (0.058 ± 0.008 spines/μm), compared to wildtype mice (Figure 1h). Therefore, we could attribute the constant loss of total spines to a diminished formation of new spines caused by tau transgene expression.

Homozygous P301S Tau mice show age-dependent neurodegeneration – especially in the brainstem [14], but also in superficial cortical layers, where mainly GABAergic interneurons are affected [21]. In order to determine whether YFP-expressing cortical neurons get lost, we followed the fate of layer V neurons’ apical dendritic trees as well as layer II/III neuronal somata in 4-month-old P301S Tau mice. However, in a time period of up to 6 weeks we did not detect the disappearance of any dendritic branches or the loss of a single neuron (analysis of 199 cells in 5 mice; data not shown).

### Morphological spine alterations in P301S tau mice

**Figure 2:**
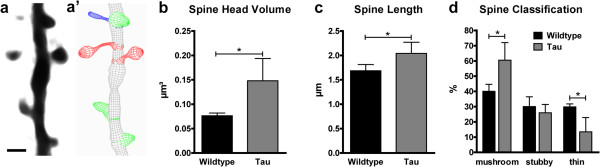
**Tau transgene expression affects dendritic spine morphology. a**-**a’ ***In vivo* imaged dendritic spines (**a** maximum intensity projection) were reconstructed (**a’**, 3D wireframe graphics) and classified into mushroom (red), stubby (green), and thin spines (blue) based on morphological parameters using Imaris software. Scale bar: 2 μm. **b**-**d** In P301S Tau mice, the spine head volume was enlarged **(b)** and the spine length was increased **(c)**, causing a shift in the fractions of morphological spine classes **(d)**. Presented are means ± SD of 3–4 mice; 130–167 spines on 10 dendrites per group; 32–45 spines on 2–4 dendrites per mouse. Unpaired *t* test of log-transformed data **(b)**, unpaired *t* test **(c, d)**; * p < 0.05.

Since dendritic spine function and morphology are strongly correlated, we performed three-dimensional reconstructions of the *in vivo* imaged dendritic spines to compare morphological parameters (Figure 2a-a’). Thereby, we found the spines of P301S Tau mice to be strongly enlarged in spine head volume (0.15 ± 0.05 μm^3^ compared to 0.08 ± 0.01 μm^3^ in wildtype; Figure 2b), accompanied by a smaller increase in spine length (2.04 ± 0.14 μm compared to 1.69 ± 0.13 μm in wildtype; Figure 2c). Moreover, we classified the spines according to their length, maximum head width, mean and minimum neck width into thin, stubby, and mushroom spines. In P301S Tau mice, the fraction of thin spines was strongly reduced (15.39 ± 10.43 compared to 28.23 ± 7.42 spines/μm in wildtype), while we measured a gain in mushroom spines (58.63 ± 16.96 compared to 39.58 ± 7.92 spines/μm in wildtype). The stubby spine fraction, however, was not significantly changed (Figure 2d).

### Cortical spine impairments in the absence of NFTs

**Figure 3:**
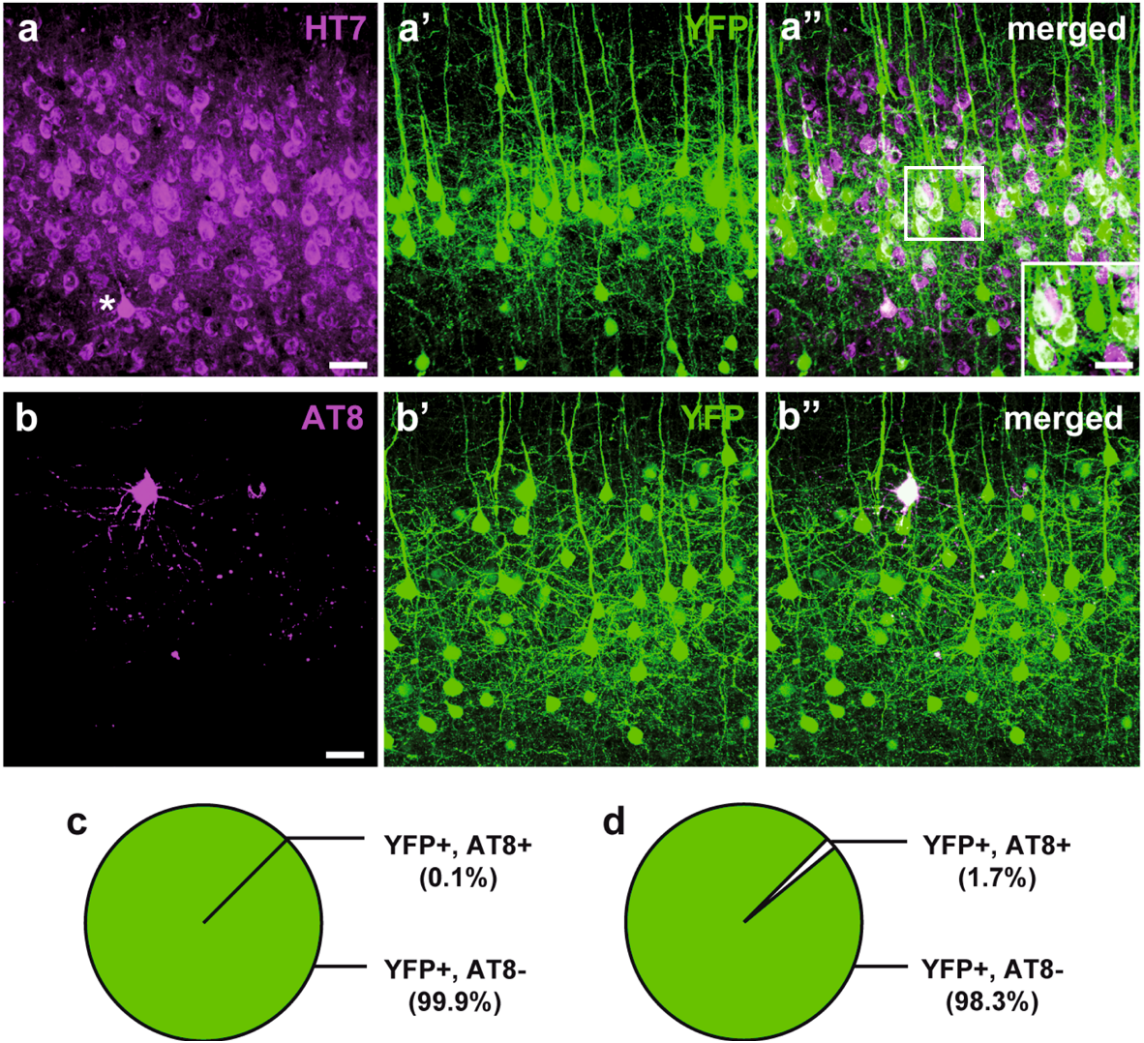
**YFP-expressing layer V neurons are free of hyperphosphorylated tau. a**-**a”** Immunohistochemical labeling of human tau (antibody HT7, magenta, **a**) in the somatosensory cortex of a 4-month-old P301S Tau x YFP-H mouse. One cell filled with densely accumulated somatodendritic tau is marked by an asterix. YFP-expressing layer V pyramidal neurons (green, **a’**) bearing the tau transgene show white cytosol labeling on the merged image (**a”**; inset). **b**-**b”** Staining of hyperphosphorylated tau (antibody AT8, magenta, **b**) in the somatosensory cortex of a 4-month-old P301S Tau x YFP-H mouse revealed only very few YFP-expressing layer V neurons (green; **b’**) to contain AT8-positive tau (white cell in **b”**). **c**-**d** Pie charts of the fractions of YFP-expressing (YFP+) layer V neurons containing (AT8+) or lacking (AT8-) AT8-positive hyperphosphorylated tau in 4- **(c)** and 6-month-old **(d)** P301S Tau mice. Presented are means ± SD of 5–6 mice per group; 1983–2433 cells in 0.32-0.39 mm^3^ tissue. **a**-**a”** and **b**-**b”** show maximum intensity projections of 50 μm z-stacks. Scale bars: 50 μm; inset: 30 μm.

In search for the causative pathological process underlying the described spine alterations in P301S Tau mice, we focused on tau-pathology in YFP-expressing cortical layer V neurons, the apical tufted dendrites of which we imaged. In the transgenic mice both YFP and the mutant human tau are expressed in neuronal subsets under the control of the murine *thy1*-promoter. Expression patterns of the transgenes can vary nevertheless, depending on stochastic factors upon transgene insertion. To verify if the dendrites imaged *in vivo* belonged to neurons expressing the human tau transgene, we performed immunohistochemical labelling for human tau on brain slices (antibody HT7; Figure 3a). Correlating the HT7-staining with the YFP-expression (Figure 3a’), we found most of the YFP-positive layer V neurons to show at least weak somatic HT7 immunoreactivity (Figure 3a”). Despite the broad expression of both proteins in the neocortex, it is not possible to verify that all of the analyzed neurons did express the tau transgene.

Homozygous P301S Tau mice develop severe tau-pathology with abundant filaments of hyperphosphorylated tau in the cerebral cortex already at young ages [14,21]. Hence, we expected many of the YFP-expressing layer V neurons to contain pre-tangle or tangle-like tau-aggregates. In search for a fluorescent, brain-permeable dye for *in vivo* labelling of these tau-deposits, we tested the amyloid-binding Congo red derivative FSB ((trans,trans)-1-fluoro-2,5-bis(3-hydroxycarbonyl-4-hydroxy)styrylbenzene) [29,30], which stains filamentous tau in the spinal cord and retina of P301S Tau mice, as shown earlier by other groups and us [19,31,32]. Applying the same method we did, however, not succeed in labelling any intracellular tau filaments in the cerebral cortex with FSB, detectable by *in vivo* imaging. Analysis of brain sections from FSB-injected mice revealed many labelled cells in the brain stem though, serving as a positive control. To further exclude the presence of NFTs in superficial cortical layers, we also tested thioflavin S. This well-established amyloid-binding fluorophore marks NFTs, such as in the brain of the rTg4510 tauopathy mouse model [9,33]. Since fluorescence spectra of YFP and thioflavin S largely overlap, we used P301S Tau mice lacking the YFP-transgene to apply thioflavin S according to the published protocol [33]. Again, no specific signal of the dye could be detected. Even 6-month-old mice, at the stage when the animals die as a consequence of the tau-pathology in the brain stem, were bare of FSB- or thioflavin S-labelled cells in the accessible cortical regions.

In default of an *in vivo* dye, we stained brain sections for hyperphosphorylated tau using the antibody AT8 (Figuire 3b-b”). To our surprise, we found AT8-positive tau only in a marginal fraction of YFP-expressing layer V neurons in 4-month-old P301S Tau mice (0.12 ± 0.13%, i.e. 3 of 2433 YFP + cells were AT8+; furthermore, 19 AT8+ cells did not express YFP (data not shown); Figure 3c), and even in 6-month-old mice (1.69 ± 2.87%, i.e. 34 of 1983 YFP + cells were AT8+; furthermore, 146 AT8+ cells did not express YFP (data not shown); Figure 3d). Hence, we conclude that the neurons imaged *in vivo* most likely did not contain detectable amounts of somatodendritic hyperphosphorylated tau nor NFTs which could account for the observed spine abnormalities.

### Immunohistochemical stainings do not reveal tau in cortical spines

**Figure 4:**
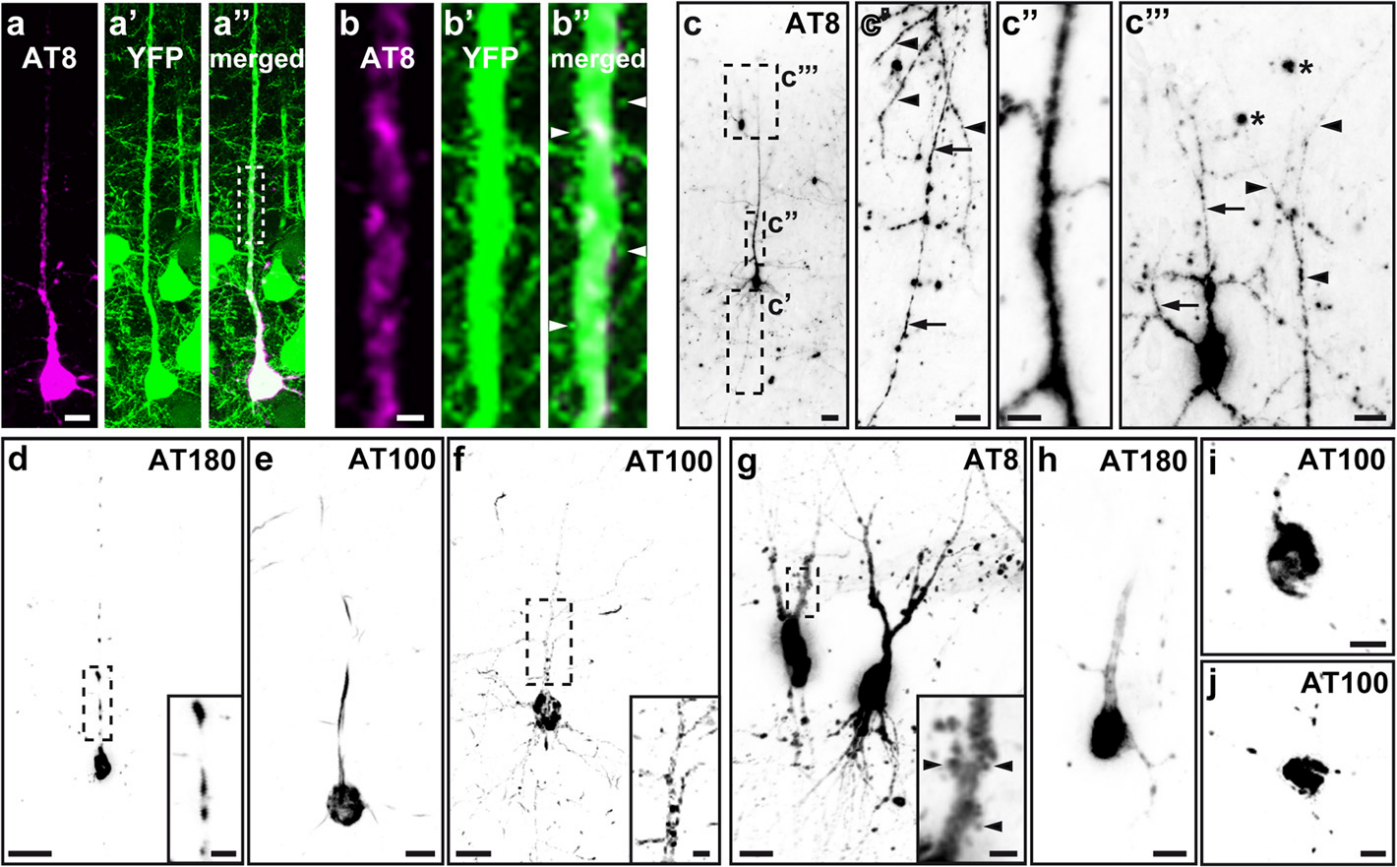
**Failure to detect tau in cortical dendritic spines by means of immunohistochemistry.** Immunohistochemical labeling of hyperphosphorylated tau (antibodies AT8, AT100, and AT180) in cortical layer V pyramidal neurons **(a-f)** and hippocampal CA3 neurons **(g-j)**. **a**-**a”** YFP-expressing neuron, filled with somatodendritic AT8-positive tau. The marked section of the apical dendrite (dashed rectangle in **a”**) is shown in higher magnification in **b**-**b”**, demonstrating that the protruding YFP-positive dendritic spines (exemplarily marked by arrowheads) are bare of hyperphosphorylated tau. **c** Example of a YFP-negative layer V neuron (**c’**-**c”’**, magnifications of the regions marked by dashed rectangles), containing AT8-positive tau in its soma, axon (arrows in **c’**), basal dendrites (arrowheads in **c’**), apical dendritic shaft **(c”)**, and higher-order distal dendritic braches (arrowheads in **c”’**). An adjacent AT8-positive layer II/III neuron shows a similar punctate pattern of hyperphosphorylated tau in its dendritic filaments (arrows in **c”’**), not to be mistaken for emanating spines. AT8-labeling is also found irregularly in the neuropil, for example in neuritic dystrophies (asterisks in **c”’**). Different from the cortex, in hippocampal CA3 neurons, AT8-positive hyperphosphorylated tau was frequently found in spiny protrusions (inset in **g**, arrows). Images show maximum intensity projections. In **c**-**j**, images were inverted for contrast enhancement. Scale bars: 2 μm **(b)**, 5 μm (**c”** & insets **d**, **f** &**g**), 10 μm **(a, c’, c”’, e, h-j)**, 20 μm **(c, d, f & g)**.

Since there is recent evidence that mislocalization of hyperphosphorylated tau to dendritic spines leads to synaptic impairments [5], we next aimed to look for tau in spines of cortical layer V neurons. Quantitative biochemical methods such as protein-detection in synaptosome- and PSD-fractions cannot detect alterations in specific populations of neurons and are therefore unable to to distinguish broad systemic effects from more subtle changes in neuronal subpopulations. Therefore, we decided to perform immunohistochemical stainings for tau on fixed brain slices of the cortical region analyzed *in vivo* using the phosphorylation-dependent antibodies AT8, AT100, and AT180 (Figure 4a-f).

We found AT8-positive hyperphosphorylated tau in the somatodendritic and axonal compartments of several neurons, as well as in neuritic dystrophies and neuropil threads, but never in dendritic spines (Figure 4a-c”’). The sparse labeling with AT100 and AT180 was usually restricted to the soma and the main shaft of the arising apical dendrite (Figure 4d-e), in a few cases also staining higher-order distal dendritic branches in a punctate pattern (Figure 4f). Unlike the phospho-tau stains, a large number of neurons were labelled with the human tau specific antibody HT7, congruent to the pan-neuronal expression of the transgene (Figure 3a). Only a few cells, however, showed neuritic staining and in none of those dendritic spines were stained.

Also after application of an alternative brain tissue fixation technique using Bouin solution, which is supposed to improve tau detection [12], (phospho-)tau could not be observed in cortical dendritic spines. These results were furthermore affirmed by DAB-stainings of conventional paraffin sections, thus avoiding impairments of antibody penetration due to section thickness. The usage of enzyme-mediated antibody detection provides strong signal enhancement, enabling tau localization even in very fine structures like axons or thin dendritic branches (Figure 4c’, c”’). By analyzing YFP-negative cells (Figure 4c-j), we also excluded detractions of antibody-binding by the presence of YFP in the postsynaptic compartment. Yet, when analyzing different brain regions like hippocampal CA3 (Figure 4g-j), AT8-positive tau was clearly localized in neuronal spiny protrusions (inset Figure 4g). AT100- and AT180-labeling was again less pronounced (Figure 4h-j), resembling the cortical staining pattern. These findings hint at distinctive pathological mechanisms, leading to brain region and neuronal subtype specific tau mislocalization in tauopathies.

## Discussion and conclusions

### Dendritic spine abnormalities in tauopathy mouse models

The generation of dozens of tau transgenic mouse models during the past years (reviewed in [34,35]) has facilitated studying the potential role of tau in neurodegenerative diseases (reviewed in [2,15]). However, with regard to synaptic density and dendritic spine morphology, inconsistent effects of tau on synapses have been described, strongly depending on the tauopathy model, the stage of the disease and the brain region analyzed [9,10,12,13]. All these approaches were performed *ex vivo*, therefore missing kinetic spine data which can only be obtained by *in vivo* imaging as presented in our study.

Dendritic spines are not rigid structures but rather bear a strong potential for morphological plasticity, thus enabling neurons to modify their synaptic interconnections, the correlates of learning and memory [36]. While the majority of spines is stable, a small fraction is permanently retracted or newly formed, even in the adult brain [20]. Underlying changes in dendritic actin filaments, modifying spine morphology, can occur on time scales from seconds to hours [37]. Changes in the total number of spines such as a reduced spine density can therefore result from either a loss of persistent spines, a diminished fraction of new spines or an enhanced fraction of retracted spines.

We measured a reduced spine density on apical tuft dendrites of cortical layer V pyramidal neurons in 4-month-old homozygous P301S Tau mice which further decreased during the two weeks imaging period. Obtaining kinetic data on single spine level, this was attributed to a diminished density of gained spines, while the stable (respectively persistent) and lost spine densities were largely unaffected compared to wildtype mice. The remaining spines underwent morphological reorganization: They were longer and the head volume was strongly enlarged, thus increasing the mushroom spine fraction at the cost of the thin spines.

Numerous studies have shown that spine volume is proportional to the size of the postsynaptic density and the AMPA (α-amino-3-hydroxy-5-methyl-4-isoxazole propionic acid) receptor content (reviewed in [38]). Thus, we propose that the observed spine remodelling displays a compensatory mechanism for the loss of total spine number, thereby strengthening the remaining synaptic contacts. Moreover, the decrease in gained spines is morphologically reflected in the diminished fraction of thin spines, which commonly correspond to non-synaptic transient precursors of the larger established spines [39,40].

Previous studies on tauopathy mouse models have obtained divergent results with regard to dendritic spines: While in most models, the spine density was found to be reduced [8-10], in others an increased [12] or unchanged spine density [13] was observed. Direct comparison of the results is hindered by both genetic differences of the mouse models used as well as specific properties of the diverse neuronal populations analyzed. However, detrimental effects of abnormal tau were found in most studies, thereby causing a loss of dendritic spines. Our study adds the important finding that expression of P301S mutant human tau impairs spine turnover and morphology, leading to a net loss of spines, accompanied by structural reorganization.

### Mislocalization of hyperphosphorylated tau to spines

It was reported recently that tau localization to dendritic spines mediates synaptic dysfunction in FTDP-17 and AD mouse models [5,6]. This was shown mainly by analyzing cultured hippocampal neurons of mice expressing P301L mutated, truncated, or wildtype human tau (lines rTg4510, pR5, Δtau74, and rTg21221). Pre- and postsynaptic accumulations of hyperphosphorylated tau in the hippocampus have also been shown for other tau transgenic mice (lines Tet-hTauP301L, 3xTg-AD, and PS19) by means of electron microscopy [10,41,42]. Moreover, tau was found to be associated to PSD95 in forebrain extracts of rTg4510 and rTg21221 mice [40] and present in a few dendritic spines of cortical neurons in rTg4510 mice [8].

When we investigated brain slices of P301S Tau mice utilizing immunohistochemistry, hyperphosphorylated tau could also clearly be located in dendritic spiny protrusions of CA3 hippocampal neurons. However, we never detected tau in dendritic spines of pyramidal neurons in the cortical regions accessible for *in vivo* imaging. The protein was even absent from spines of neurons in which the somatodendritic compartment was almost completely filled with filamentous hyperphosphorylated tau.

The brain region specific mislocalization of hyperphosphorylated tau to dendritic spines in the tauopathy mouse model analyzed here is supported by very similar findings in AD patients. There, only the thorny excrescences of CA3 hippocampal neurons contain hyperphosphorylated tau, but not dendritic spines in other fields of the hippocampal formation or the adjacent cortex [23,24]. Unlike dendritic spines of cortical pyramidal neurons, which have an actin-based cytoskeleton, the CA3 thorny excrescences occasionally contain microtubules [43,44]. This explains the presence of microtubule-associated proteins like tau inside the spiny protrusions. In conclusion, we suggest different mechanisms underlying synaptic impairment in tauopathies, depending on specific characteristics of the affected neuronal populations. Moreover, these accumulations of hyperphosphorylated tau in hippocampal postsynapses could also cause dysfunctions of the local or further interconnected neuronal networks. Thus, impaired hippocampal signaling might contribute indirectly to cortical spine pathology.

### Axonal pathology and local network dysfunction causing spine impairments

Methodical restrictions make it nearly impossible to synchronously follow the fate of single dendritic spines and their presynaptic counterparts (i. e. axonal boutons) *in vivo*. Hence, with our kinetic data we cannot clarify if the loss of spines is a primary or secondary event, caused by axonal pathology which is known to be very prominent in tauopathies [45-47]. Given the lack of tau transgene expression in a fraction of YFP-positive layer V neurons makes it even more likely that presynaptic failure partially accounts for the dendritic spine impairments.

Moreover, a severe neuron loss in superficial cortical layers, mainly affecting GABAergic interneurons, was reported for homozygous P301S Tau mice already at the age of 3 months [21]. Since those inhibitory interneurons also form connections with pyramidal cells [48], a decline in inhibition might lead to a compensatory reduction of excitatory synapses. Neurodegeneration in P301S Tau mice, which could only partially be correlated to intracellular tau-deposits, is also accompanied by inflammatory responses [17]. Microglia and astrocytes are known to be critically involved in healthy brain homeostasis and synaptic maintenance [49-51]. Therefore, the induction of inflammatory processes can have significant implications on dendritic spine plasticity, thus preventing the emergence of new spines or causing morphological alterations. Summarized, the observed changes in dendritic spines might be an indirect effect, caused by axonal pathology or local network dysfunction.

### In search for the toxic tau species

We demonstrated in this study, that dendritic spine loss and remodelling in cortical neurons of P301S Tau mice occur in the absence of hyperphosphorylated tau accumulation. This is in line with other studies showing that tau-aggregates and even NFTs are not sufficient to cause cognitive decline or neurodegeneration: In a reversible tauopathy mouse model, memory function recovered after tau transgene suppression despite continuing NFT build-up [52]. Furthermore, structural and functional neuronal changes in different tauopathy mice are independent of the presence of NFTs [9,10], and accumulation of insoluble tau does not necessarily affect spine density and morphology [53]. On the other hand, there is strong evidence for the existence of neurotoxic prefibrillar tau dimers and higher order oligomeric aggregates in tauopathies such as AD [54-56]. Also, a dendritic function of tau monomers was suggested to mediate excitotoxicity in AD mouse models [6]. Therefore, we argue for prefibrillar tau as the toxic species in tau-dependent neurodegenerative diseases. Further studies will be needed to clarify the specific pathogenic mechanism and to finally find ways of disease prevention, arrest, or even cure.

## Competing interests

The authors declare that they have no conflict of interests.

## Authors’ contributions

NAH conceived experiments and performed experiments, statistical analysis, manuscript drafting and manuscript assembly. MMD participated in statistical analysis and manuscript drafting. SB provided technical support. MG contributed materials and provided technical support. JH conceived experiments and participated in manuscript drafting. All authors read and approved the final manuscript.
